# Sodium butyrate attenuates D-galactose-induced cellular senescence in WI-38 fibroblasts *in vitro* via modulation of HDAC activity, Nrf2/ARE pathway, and p53/p21/p16 signaling

**DOI:** 10.3389/fphys.2026.1811143

**Published:** 2026-06-01

**Authors:** Kangping Song

**Affiliations:** School of Medicine, Johns Hopkins University, Baltimore, MD, United States

**Keywords:** cellular senescence, epigenetic regulation, HDAC, Nrf2, p53/p21/p16, sodium butyrate

## Abstract

Cellular senescence is closely associated with various age-related diseases. Sodium butyrate (NaB), a short-chain fatty acid and histone deacetylase (HDAC) inhibitor, exhibits potential anti-aging properties; however, its precise mechanisms remain unclear. This study aimed to investigate the protective effects of NaB against D-galactose (D-gal)-induced senescence in WI-38 human fibroblasts and elucidate the underlying mechanisms. WI-38 cells were co-treated with D-gal and NaB simultaneously to investigate whether NaB could mitigate D-gal-induced senescent changes. Senescence phenotypes were assessed by SA-β-Gal staining, ROS detection, and ELISA. Western blotting and chromatin immunoprecipitation-quantitative PCR (ChIP-qPCR) were performed to evaluate signaling pathways and histone acetylation, while siRNA knockdown validated Nrf2 function. NaB dose-dependently reduced SA-β-Gal-positive cells, ROS levels, and MDA content, while restoring SOD and GSH-Px activities and suppressing IL-6 and IL-1β secretion. Mechanistically, NaB promoted Nrf2 nuclear translocation by downregulating Keap1, thereby upregulating HO-1 and NQO1 expression. NaB also inhibited p53 phosphorylation and reduced p21 and p16 expression at both the mRNA and protein levels, as confirmed by RT-qPCR and Western blotting. Furthermore, NaB suppressed HDAC activity and restored H3K9ac and H3K27ac levels. Nrf2 knockdown reversed NaB’s protective effects. ChIP-qPCR revealed that NaB restored H3K9ac enrichment at p21 and p16 promoters. These results indicate that NaB may attenuate D-gal-induced cellular senescence through coordinated modulation of the Nrf2/ARE pathway, p53/p21/p16 signaling, and HDAC-mediated epigenetic regulation. These findings provide preliminary evidence supporting NaB as a candidate modulator of aging-associated cellular processes, warranting further validation *in vivo*.

## Graphical Abstract

**Figure:**
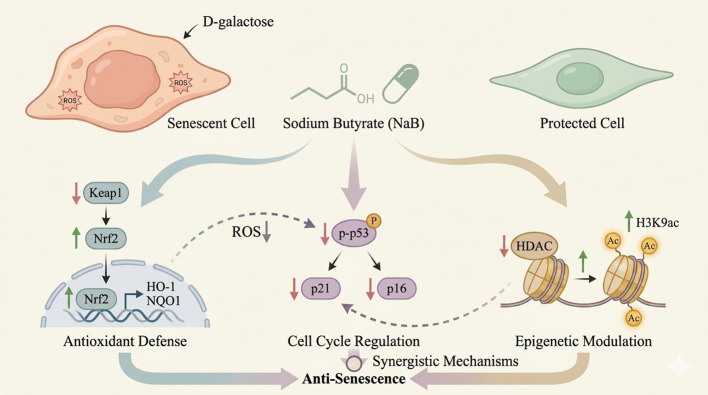
Illustrating the anti-senescence mechanisms of NaB.

## 1 Introduction

Cellular senescence is a state of stable cell cycle arrest induced by various factors, including telomere shortening, DNA damage, oxidative stress, and oncogene activation (Hayflick and Moorhead, 1961). Although senescent cells lose their proliferative capacity, they remain metabolically active and secrete substantial amounts of pro-inflammatory cytokines, chemokines, growth factors, and matrix metalloproteinases, collectively known as the senescence-associated secretory phenotype (SASP) (Coppé et al., 2010). Through paracrine signaling, SASP can induce senescence in neighboring cells, exacerbate inflammation in the tissue microenvironment, and thereby contribute to aging-related physiological decline and the pathogenesis of age-related diseases such as atherosclerosis, type 2 diabetes, and neurodegenerative disorders (Baker et al., 2011; Childs et al., 2015). Consequently, elucidating the molecular mechanisms that govern cellular senescence is of fundamental importance for understanding the physiological process of aging.

The p53/p21WAF1/p16INK4a signaling pathway constitutes a central molecular mechanism governing cellular senescence (Huang et al., 2024). Upon oxidative stress or DNA damage, p53 undergoes phosphorylation and activation, leading to upregulation of its downstream target p21WAF1 (CDKN1A), which inhibits cyclin-dependent kinase (CDK) activity and induces G1/S phase arrest (Yan et al., 2024). Additionally, p16INK4a (CDKN2A), another key senescence marker, can inhibit CDK4/6 activity independently of the p53 pathway, maintaining the retinoblastoma protein (Rb) in a hypophosphorylated state and thereby reinforcing cell cycle arrest (Serrano et al., 1993). Sustained high expression of p21 and p16 has been shown to be critical for the establishment of an irreversible senescent state (López-Otín et al., 2023).

Oxidative stress serves as a major driver of cellular senescence (Davalli et al., 2016). Excessive accumulation of reactive oxygen species (ROS) can directly damage DNA, proteins, and lipids, thereby activating the p53/p21 pathway (Muthamil et al., 2024). Nuclear factor erythroid 2-related factor 2 (Nrf2) is a master transcription factor that coordinates cellular defense against oxidative stress. Under basal conditions, Nrf2 is bound by Kelch-like ECH-associated protein 1 (Keap1) and targeted for ubiquitin-mediated degradation. Upon oxidative challenge, Nrf2 dissociates from Keap1, translocates to the nucleus, and promotes transcription of downstream antioxidant genes such as heme oxygenase-1 (HO-1) and NAD(P)H quinone oxidoreductase 1 (NQO1) (Ngo and Duennwald, 2022). Notably, Nrf2 activity declines with age, whereas pharmacological or genetic modulation of the Nrf2 pathway has been shown to influence cellular and organismal aging (Kubben et al., 2016; Medoro et al., 2024).

Epigenetic regulation has emerged as an important contributor to cellular senescence. Histone deacetylases (HDACs) remove acetyl groups from lysine residues on histones, leading to chromatin condensation and transcriptional repression; notably, HDAC activity has been directly implicated in the regulation of cellular senescence (Lee et al., 2022). Studies have revealed that HDAC activity is elevated in senescent cells, resulting in reduced acetylation at histone H3 lysine 9 (H3K9ac) and lysine 27 (H3K27ac) and consequent downregulation of antioxidant and cytoprotective genes (Sen et al., 2016). Importantly, HDAC inhibitors have been shown to modulate aging phenotypes by restoring histone acetylation and reactivating silenced genes (McIntyre et al., 2019).

Sodium butyrate (NaB) is a naturally occurring short-chain fatty acid produced primarily through gut microbial fermentation of dietary fiber (Louis and Flint, 2017). As a broad-spectrum class I/II HDAC inhibitor, NaB modulates gene expression by increasing histone acetylation and has been implicated in the regulation of inflammatory responses, cellular proliferation, and barrier function (Davie, 2003; Liu et al., 2018). Recent evidence suggests that NaB may influence aging-related processes, extending lifespan in model organisms and ameliorating age-related phenotypes (Walsh et al., 2015).

The D-galactose (D-gal)-induced senescence model is widely used to study aging at both cellular and organismal levels (Shwe et al., 2018). High concentrations of D-gal generate excessive ROS via galactose oxidase activity, leading to oxidative damage that mimics key aspects of the natural aging process (Ho et al., 2003). This model has been extensively employed in mechanistic investigations of aging-related processes.

While the individual effects of NaB on Nrf2 activation and HDAC inhibition have been separately reported, no prior study has systematically integrated these mechanisms within a unified senescence model and validated their functional interdependence. Specifically, the causal role of Nrf2 in NaB’s anti-senescence activity has not been established via loss-of-function experiments, and the epigenetic regulation of senescence-associated gene promoters (p21 and p16) by NaB-mediated histone acetylation has not been directly examined by ChIP-qPCR. The present study addresses these gaps by providing an integrated mechanistic analysis linking Nrf2 signaling, p53 pathway suppression, and HDAC-dependent chromatin remodeling in a single cellular senescence model. Based on this background, the present study utilized a D-gal-induced senescence model in WI-38 human embryonic lung fibroblasts to systematically examine the effects of NaB on cellular senescence and elucidate the underlying molecular mechanisms from a physiological perspective. Specifically, we investigated how NaB modulates oxidative stress, the Nrf2/ARE antioxidant pathway, the p53/p21/p16 senescence axis, and HDAC-mediated epigenetic regulation. By integrating these mechanistic analyses, this study aimed to provide insights into the physiological regulation of cellular senescence and the potential role of NaB as a modulator of aging-related cellular processes.

## 2 Materials and methods

### 2.1 Main reagents and instruments

WI-38 human embryonic lung fibroblasts were obtained from the American Type Culture Collection (ATCC, Manassas, VA, USA). D-Galactose and sodium butyrate were supplied by Sigma-Aldrich. The CCK-8 kit and SA-β-Gal staining kit were from Dojindo and Cell Signaling Technology, respectively. Primary antibodies against Keap1, HO-1, Nrf2, NQO1, p53, p21, p16, H3K9ac, H3K27ac, and β-actin were obtained from Cell Signaling Technology or Abcam. Commercial kits for MDA, SOD, and GSH-Px measurements were from Nanjing Jiancheng Bioengineering Institute. The HDAC activity assay kit was purchased from Abcam. siRNA targeting Nrf2 and negative control siRNA were synthesized by GenePharma. Flow cytometry was performed on a FACSCanto II system (BD Biosciences).

### 2.2 Cell culture and treatment

WI-38 fibroblasts were propagated in high-glucose DMEM containing 10% heat-inactivated FBS and antibiotics (100 U/mL penicillin and 100 μg/mL streptomycin). Cultures were maintained under standard conditions (37°C, 5% CO_2_, humidified atmosphere). Subculturing was performed at 80–90% confluence using 0.25% trypsin-EDTA digestion. Experiments utilized cells in the logarithmic growth phase at passages 5–15 (Wang et al., 2022). To minimize passage-dependent variability, all experiments were performed using cells within a narrow passage range (passages 8–12), and cells from different passage numbers were not mixed within the same experiment. Cells were verified to maintain consistent morphology and proliferative capacity prior to use.

### 2.3 Cell viability assay

Cellular viability was quantified using the CCK-8 method. WI-38 cells were plated in 96-well plates (5 × 10³ cells/well) and incubated overnight for attachment. For NaB cytotoxicity evaluation, cells received NaB treatment at varying concentrations (0, 0.25, 0.5, 1, 2, 4, and 8 mM) for 24 h. To optimize D-gal-induced senescence parameters, cells were challenged with D-gal (0, 20, 40, 60, 80, and 100 mM) for either 48 or 72 h.

Following treatment completion, culture medium was discarded and substituted with serum-free medium supplemented with 10% CCK-8 reagent (100 μL/well). After a 2 h incubation at 37°C protected from light, optical density was recorded at 450 nm using a SpectraMax M5 microplate reader (Molecular Devices, San Jose, CA, USA). Viability percentages were determined using the formula:

Cell viability (%) = (OD_experimental_ - OD_blank_)/(OD_control_ - OD_blank_) × 100%.

The blank contained medium plus CCK–8 reagent only, and the control consisted of untreated cells. Each condition was assayed in six replicate wells, and all experiments were performed in triplicate.

### 2.4 Senescence-associated β-galactosidase staining

Cells were washed with phosphate–buffered saline (PBS) and fixed for 15 min at room temperature. Following fixation, cells were incubated overnight at 37 °C in a CO_2_–free incubator with freshly prepared SA–β–Gal staining working solution. SA–β–Gal–positive cells, identified by the presence of blue cytoplasmic granules, were visualized using an inverted microscope. For each group, five randomly selected fields were examined, with a minimum of 200 cells counted per sample. The percentage of SA–β–Gal positive cells was then calculated (Kudlova et al., 2022).

### 2.5 ROS detection

Intracellular ROS levels were assessed using the fluorescent probe 2′,7′-dichlorofluorescein diacetate (H2DCF–DA) (Geng et al., 2023). Cells not loaded with H2DCF–DA served as the negative control. Following treatment, cells were collected, washed with phosphate–buffered saline (PBS), and incubated with 10 μM H2DCF–DA at 37 °C for 30 min in the dark. After incubation, cells were washed again with PBS to remove residual probe. Fluorescence intensity was then measured by flow cytometry to quantify intracellular ROS levels. For data acquisition, a minimum of 10,000 events were recorded per sample. Debris was excluded based on forward scatter (FSC) and side scatter (SSC) gating, and doublets were excluded using FSC-H versus FSC-A plots. Cells stained with H2DCF-DA were compared against an unstained control to establish the baseline fluorescence threshold and define the ROS-positive population. Cell viability was confirmed by propidium iodide (PI) exclusion prior to H2DCF-DA staining to ensure that fluorescence signals reflected live-cell ROS levels.

### 2.6 Detection of oxidative stress and inflammatory markers

Following treatment, supernatants were harvested by centrifugation (1,000 × g, 10 min, 4°C) and stored at -80°C for cytokine analysis. Cells were rinsed with ice-cold PBS, lysed on ice for 30 min, and centrifuged (12,000 × g, 10 min, 4°C) to obtain lysates for oxidative stress assessment. IL-6 and IL-1β levels were quantified by ELISA. MDA content, SOD activity, and GSH-Px activity were measured using commercial kits and normalized to total protein. All assays followed manufacturer’s protocols, with concentrations derived from standard curves.

### 2.7 Western blot analysis

Cells were harvested, rinsed twice with ice-cold PBS, and lysed in RIPA buffer supplemented with protease/phosphatase inhibitors for 30 min on ice with intermittent vortexing. After centrifugation (12,000 × g, 15 min, 4°C), supernatants containing total protein were collected and stored at –80°C. Nuclear and cytoplasmic fractions were prepared using a commercial extraction kit. Protein concentrations were measured by BCA assay. Samples (20-40 μg) were mixed with loading buffer, heat-denatured, resolved by SDS-PAGE, and transferred onto PVDF membranes. Following blocking with 5% skim milk in TBST (1 h, room temperature), membranes were probed overnight at 4°C with primary antibodies against Keap1, Nrf2, HO-1, NQO1, p53, p-p53 (Ser15), p21, p16, H3K9ac, H3K27ac, Histone H3, and β-actin (dilutions ranging from 1:1000 to 1:5000). After TBST washes, membranes were incubated with HRP-conjugated secondary antibody (1:5000, 1 h). Bands were visualized by ECL and quantified using ImageJ. Cytoplasmic proteins were normalized to β-actin; nuclear proteins were normalized to Histone H3.

### 2.8 Cellular HDAC activity fluorescence detection

HDAC activity in cells from each group was assessed using a commercially available HDAC activity fluorescence assay kit. Cells were plated in confocal dishes at an appropriate density and allowed to adhere prior to treatment according to the experimental groups. After treatment, the culture supernatant was removed and replaced with the assay working solution containing the HDAC–sensitive fluorescent substrate, followed by incubation at 37 °C in the dark for 30 min. Cells were then gently washed three times with pre–warmed phosphate–buffered saline (PBS) and kept hydrated in PBS during imaging. Fluorescence images were captured using a fluorescence microscope equipped with an FITC filter set (excitation/emission: 488/525 nm). At least three non–overlapping fields were randomly acquired per group. Green fluorescence intensity was quantified and considered to reflect relative HDAC activity.

### 2.9 siRNA transfection and Nrf2 knockdown validation

To determine whether the anti-senescence effects of NaB depend on the Nrf2 pathway, Nrf2 expression was knocked down using small interfering RNA (siRNA). siRNA targeting human NFE2L2 (Nrf2) and a negative control siRNA (si-NC) were synthesized by GenePharma (Shanghai, China). The siRNA sequences were as follows:

si-Nrf2: sense 5′-GAGUUACAGUGUCUUAAUATT-3′, antisense 5′-UAUUAAGACACUGUAACUCTT-3′

si-NC: sense 5′-UUCUCCGAACGUGUCACGUTT-3′, antisense 5′-ACGUGACACGUUCGGAGAATT-3′

WI-38 cells were seeded in 6-well plates at a density of 2 × 10^5^ cells per well and transfected at 40-60% confluence. Transfection complexes were prepared according to the Lipofectamine 3000 protocol: siRNA (final concentration 50 nM) was diluted in Opti–MEM medium, mixed with P3000 reagent, incubated at room temperature for 15 min, and added dropwise to the cells. Complete medium was replaced 6 h after transfection, and drug treatments were initiated 24 h later. Based on prior Western blot results, the most effective high–dose NaB concentration (4 mM) was selected for subsequent experiments. The following four groups were established: (1) Normal control group (Con): routine culture for 72 h; (2) D-gal model group (D-gal): 40 mM D-gal treatment for 72 h; (3) D-gal+NaB+si-NC group: transfected with negative control siRNA, then co-treated with 40 mM D-gal and 4 mM NaB for 72 h; (4) D-gal+NaB+si-Nrf2 group: transfected with Nrf2 siRNA, then co-treated with 40 mM D-gal and 4 mM NaB for 72 h. After treatment, Nrf2 and its downstream target HO–1 were detected by Western blot to confirm knockdown efficiency. In parallel, SA–β–Gal staining and flow–cytometric ROS measurement were performed to assess the impact of Nrf2 silencing on the anti–senescence activity of NaB.

### 2.10 ChIP-qPCR

To examine whether NaB modulates p21 and p16 transcription via histone acetylation, ChIP-qPCR was performed to assess H3K9ac enrichment at their promoter regions. Experiments were divided into three groups: (1) Normal control group (Con): routine culture for 72 h; (2) D-gal model group (D-gal): 40 mM D-gal treatment for 72 h; (3) D-gal+NaB group: co-treatment with 40 mM D-gal and 4 mM NaB for 72 h. The NaB concentration was selected based on Western blot results showing optimal effects at this dose. ChIP was conducted using the SimpleChIP^®^ Enzymatic Chromatin IP Kit following manufacturer’s instructions. Briefly, cells (1×10^7^) were crosslinked with 1% formaldehyde (10 min), quenched with glycine, and sonicated to yield 200–500 bp fragments. Equal amounts of chromatin were immunoprecipitated overnight at 4°C with anti-H3K9ac antibody (5 μg) or normal rabbit IgG (negative control). After incubation with Protein G magnetic beads and sequential washes, DNA was eluted, reverse-crosslinked, purified, and used for downstream analysis. Input samples (1% of total chromatin) served as normalization controls. Real-time quantitative PCR was performed to detect H3K9ac enrichment at p21 (CDKN1A) and p16 (CDKN2A) gene promoter regions.

Primer sequences were:

p21 forward 5’-GTGGCTCTGATTGGCTTTCTG-3’, reverse 5’-CTGAAAACAGGCAGCCCAAG-3’;

p16 forward 5’-CGCTAAGTGCTCGGAGTTAATA-3’, reverse 5’-GGCTGAACTTTCTGTGCTGG-3’.

qPCR reactions used SYBR Green premix with cycling conditions: 95 °C for 10 min; 95 °C (15 s), 60 °C (60 s), for 40 cycles. Three technical replicates were set for each sample. Results were calculated using the % Input method:

% Input = 2^Ct_Input - Ct_IP^ × Input dilution factor × 100% (He et al., 2021).

Each ChIP experiment was performed as an independent biological replicate (n = 3, from separately cultured cell batches), with three technical replicates (qPCR triplicates) per biological replicate. Data are reported as the mean ± SD of biological replicates.

### 2.11 RNA extraction and RT-qPCR

Total RNA was extracted from WI-38 cells using TRIzol reagent (Invitrogen) according to the manufacturer’s protocol. RNA concentration and purity were assessed using a NanoDrop spectrophotometer (Thermo Fisher Scientific); samples with an A260/A280 ratio between 1.8 and 2.0 were used for downstream analysis. Complementary DNA (cDNA) was synthesized from 1 μg of total RNA using a PrimeScript RT Reagent Kit (Takara Bio). RT-qPCR was performed using SYBR Green premix under the following cycling conditions: 95 °C for 30 s; 40 cycles of 95 °C for 5 s and 60 °C for 30 s. GAPDH served as the internal reference gene, and relative expression was calculated using the 2^-ΔΔCt^ method.

Primer sequences were:

CDKN1A (p21) forward: 5’-CATGTGGACCTGTCACTGTCTTG-3’, reverse: 5’-GCAAATCTGTCATGCTGGTCTG-3’;

CDKN2A (p16) forward: 5’-GGGTTTTCTTGGTGAAGTTCG-3’, reverse: 5’-TTGCCCATCATCATCACCTG-3’;

GAPDH forward: 5’-GTCTCCTCTGACTTCAACAGCG-3’, reverse: 5’-ACCACCCTGTTGCTGTAGCCAA-3’.

Three independent biological replicates were performed, each with three technical replicates.

### 2.12 Statistical analysis

All assays were repeated independently at least three times (n = 3 independent biological replicates, each performed on separately cultured cell batches on different days), and data are reported as mean ± SD. Data were assumed to follow a normal distribution based on the small-sample nature of the experiments; formal normality testing was not performed given the limited sample size. Sample sizes were chosen based on common practice in comparable *in vitro* senescence studies and were sufficient to detect biologically meaningful differences with the statistical tests applied. Statistical analyses were carried out with GraphPad Prism 9.0. One-way ANOVA with Tukey’s *post hoc* test was used for multiple group comparisons, while Student’s t-test was applied for two-group comparisons. P < 0.05 was considered statistically significant.

## 3 Results

### 3.1 Establishment of D-gal-induced WI-38 cell senescence model

**Figure 1:**
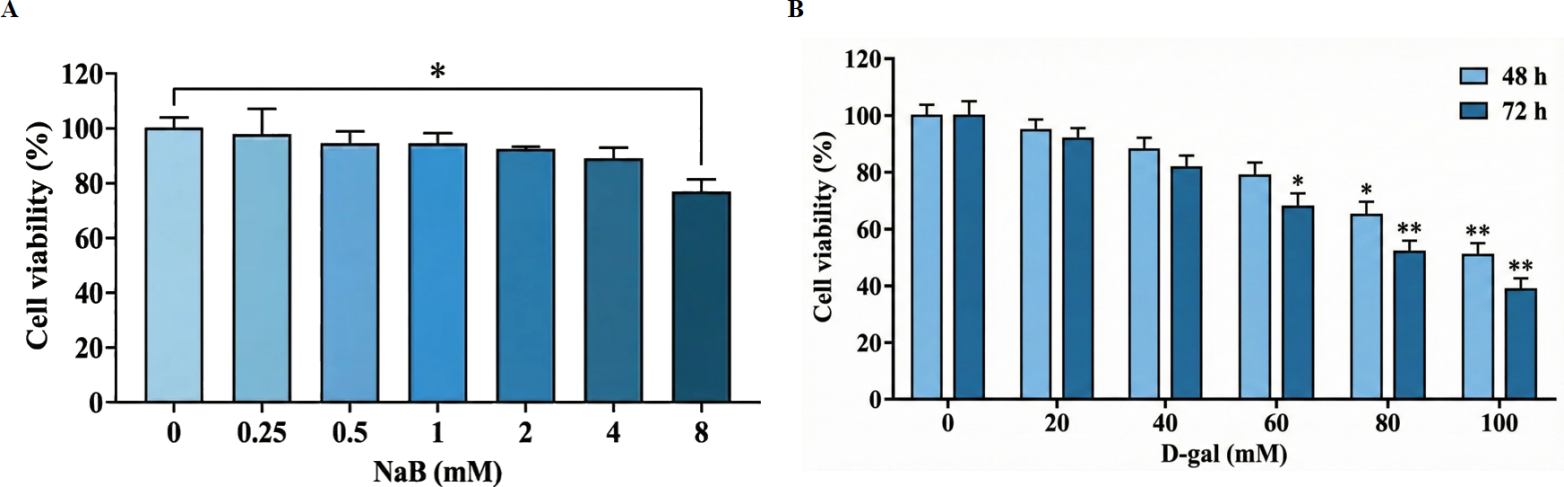
Effects of NaB and D-gal on WI-38 cell viability. **(A)** Cell viability of WI-38 cells treated with different concentrations of NaB (0–8 mM) for 24 (h). **(B)** Cell viability of WI-38 cells treated with different concentrations of D-gal (0–100 mM) for 48 h and 72 (h) Data are presented as mean ± SD (n=3). *p < 0.05, **p < 0.01 vs. control group.

To determine the non–toxic concentration range of NaB and optimal conditions for D–gal–induced senescence, cell viability was first assessed using the CCK–8 assay. As shown in **Figure 1A**, treatment with NaB at concentrations up to 4 mM did not significantly affect cell viability, which remained >85% (P > 0.05 versus control). In contrast, 8 mM NaB markedly reduced viability to 78.3 ± 5.2% (P < 0.05), indicating cytotoxicity at this higher concentration. Therefore, 0.5, 2, and 4 mM NaB were selected as low, medium, and high doses, respectively, for subsequent experiments.

To establish an appropriate senescence–induction protocol, cells were exposed to D–gal (0–100 mM) for 48 h or 72 h. D–gal decreased viability in a concentration– and time–dependent manner (**Figure 1B**). After 48 h, viability was significantly lowered at ≥60 mM D–gal (P < 0.05), and the effect was more pronounced after 72 h. Treatment with 40 mM D–gal for 72 h reduced viability to 82.3 ± 4.2% (≈18% decrease), a condition that induced substantial oxidative stress without causing excessive cell death and was therefore chosen for senescence induction. At ≥60 mM D–gal for 72 h, viability fell below 70% (P < 0.01), likely reflecting acute cytotoxicity rather than senescence. Consequently, 40 mM D–gal for 72 h was adopted as the standard senescence model.

Based on these preliminary data, cells were assigned to five groups: (1) Normal control (Con): routine culture for 72 h; (2) D–gal model (D–gal): 40 mM D–gal for 72 h; (3) D–gal + low–dose NaB (D–gal+L): co–treatment with 40 mM D–gal and 0.5 mM NaB for 72 h; (4) D–gal + medium–dose NaB (D–gal+M): co–treatment with 40 mM D–gal and 2 mM NaB for 72 h; (5) D–gal + high–dose NaB (D–gal+H): co–treatment with 40 mM D–gal and 4 mM NaB for 72 h. D–gal was maintained throughout the treatment period.

### 3.2 NaB alleviates D-gal-induced senescence in WI-38 cells

**Figure 2:**
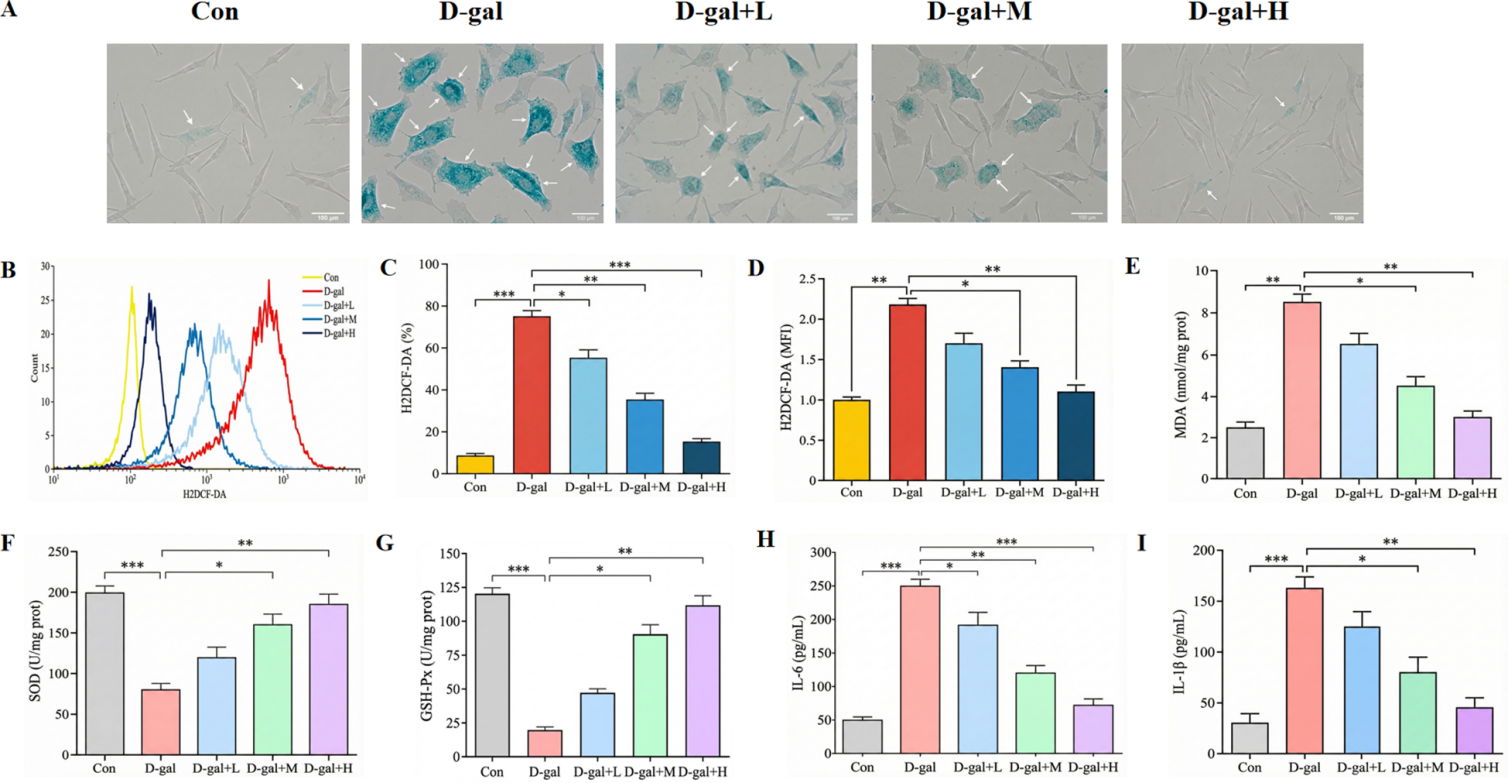
NaB attenuates D-gal-induced cellular senescence, oxidative stress, and SASP in WI-38 cells. **(A)** Representative images of SA-β-Gal staining (scale bar = 100 μm). White arrows indicate SA-β-Gal positive cells; **(B)** Flow cytometry analysis of intracellular ROS levels using H2DCF-DA probe; **(C)** Quantification of ROS-positive cell percentage; **(D)** Mean fluorescence intensity (MFI) of H2DCF-DA; **(E)** MDA content in cell lysates; **(F)** SOD activity in cell lysates; **(G)** GSH-Px activity in cell lysates; **(H)** IL-6 levels in culture supernatants measured by ELISA; **(I)** IL-1β levels in culture supernatants measured by ELISA. Data are presented as mean ± SD (n = 3). *P < 0.05, **P < 0.01, ***P < 0.001.

To evaluate the protective effects of NaB against D–gal–induced senescence, SA–β–Gal activity was first examined. As shown in **Figure 2A**, control cells displayed typical spindle–shaped morphology with minimal blue staining. In contrast, D–gal–treated cells exhibited marked flattening, increased cell size, and a significantly higher percentage of SA–β–Gal–positive cells, thereby confirming successful senescence induction. NaB co–treatment dose–dependently reduced the proportion of SA–β–Gal–positive cells, and cellular morphology in the high–dose NaB group (D–gal+H) closely resembled that of the control.

Since oxidative stress is a key driver of senescence, intracellular ROS levels were measured using the H2DCF–DA probe coupled with flow cytometry. The D–gal group showed a pronounced increase in both the percentage of ROS–positive cells (≈78%) and the mean fluorescence intensity (MFI) compared with the control (P < 0.001; **Figures 2B–D**). NaB treatment attenuated ROS accumulation in a dose–dependent manner, with the D–gal+H group displaying a ROS–positive rate near 15% and MFI values comparable to the control (P < 0.01 versus D–gal alone).

To further assess the impact of NaB on redox balance, MDA content and the activities of the antioxidant enzymes SOD and GSH–Px were determined. D–gal exposure significantly elevated MDA levels (P < 0.01) and suppressed both SOD and GSH–Px activities (P < 0.001; **Figures 2E–G**). NaB co–treatment reversed these changes in a dose–dependent fashion, and all measured parameters in the high–dose NaB group were restored to levels statistically indistinguishable from the control, indicating effective alleviation of D–gal–induced oxidative damage.

Senescent cells often exhibit a heightened secretory profile, termed the SASP. ELISA analysis revealed that D–gal treatment markedly increased the secretion of the pro–inflammatory cytokines interleukin–6 (IL–6) and interleukin–1β (IL–1β) (P <0.001 versus control; **Figures 2H, I**). NaB administration suppressed the release of both cytokines in a dose–dependent manner, with significant reductions observed in the D–gal+H group compared with the D–gal group (P < 0.01).

### 3.3 NaB activates the Keap1/Nrf2/ARE signaling pathway in D-gal-induced senescent WI-38 cells

**Figure 3:**
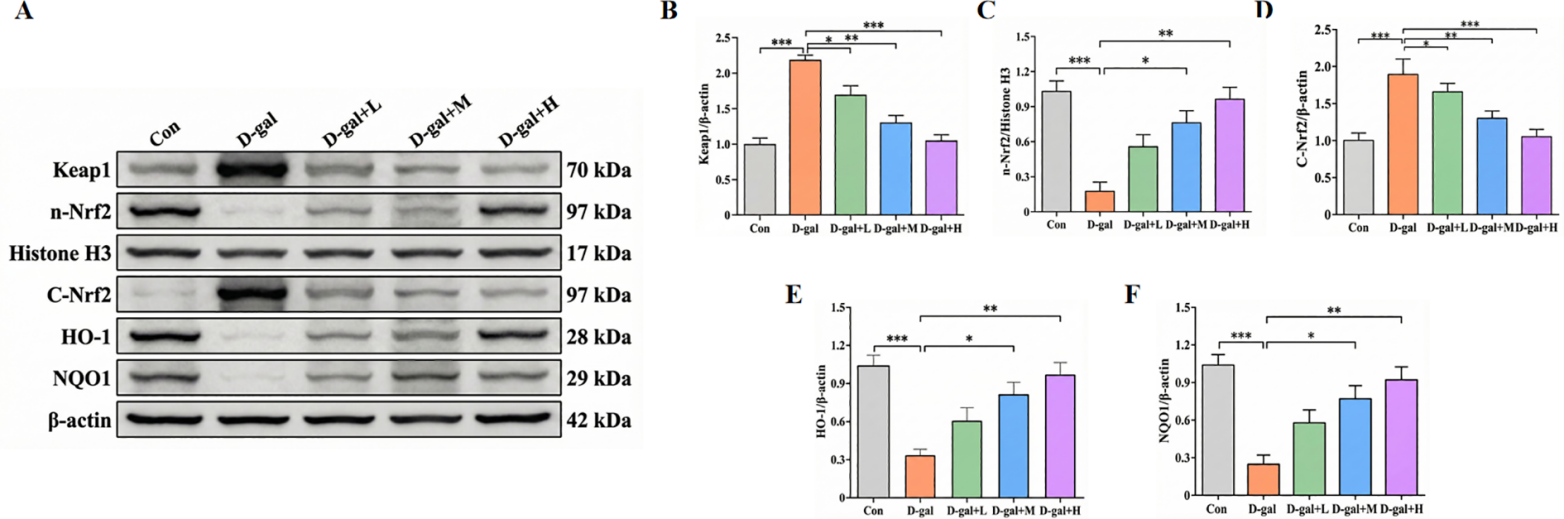
NaB activates the Keap1/Nrf2/ARE signaling pathway in D-gal-induced senescent WI-38 cells. **(A)** Representative Western blot images of Keap1, nuclear Nrf2 (n-Nrf2), cytoplasmic Nrf2 (C-Nrf2), HO-1, and NQO1. Histone H3 and β-actin served as loading controls for nuclear and total/cytoplasmic proteins, respectively. **(B)** Quantification of Keap1/β-actin. **(C)** Quantification of n-Nrf2/Histone H3. **(D)** Quantification of C-Nrf2/β-actin. **(E)** Quantification of NQO1/β-actin. **(F)** Quantification of HO-1/β-actin. Data are presented as mean ± SD (n = 3). *P < 0.05, **P < 0.01, ***P < 0.001.

Given that oxidative stress is a major driver of cellular senescence and the Nrf2/ARE pathway serves as a master regulator of antioxidant defense (Kubben et al., 2016; Ngo and Duennwald, 2022; Medoro et al., 2024), we hypothesized that NaB may exert its anti-aging effects through Nrf2 activation. To test this hypothesis, the expression of Keap1/Nrf2 pathway components was examined by Western blot. As shown in **Figures 3A–F**, D–gal treatment markedly upregulated Keap1 (P < 0.001) and increased C–Nrf2 (P < 0.001), while n–Nrf2 and its downstream targets HO–1 and NQO1 were significantly downregulated (P < 0.001). NaB co–treatment dose–dependently reduced Keap1 expression, decreased C–Nrf2, restored n–Nrf2 levels, and upregulated HO–1 and NQO1. Notably, n–Nrf2 in the high–dose NaB group was significantly higher than in the D–gal group (P < 0.01), indicating that NaB promotes Nrf2 nuclear translocation through Keap1 downregulation, leading to activation of antioxidant gene transcription.

### 3.4 NaB inhibits the p53/p21/p16 signaling pathway in D-gal-induced senescent WI-38 cells

**Figure 4:**
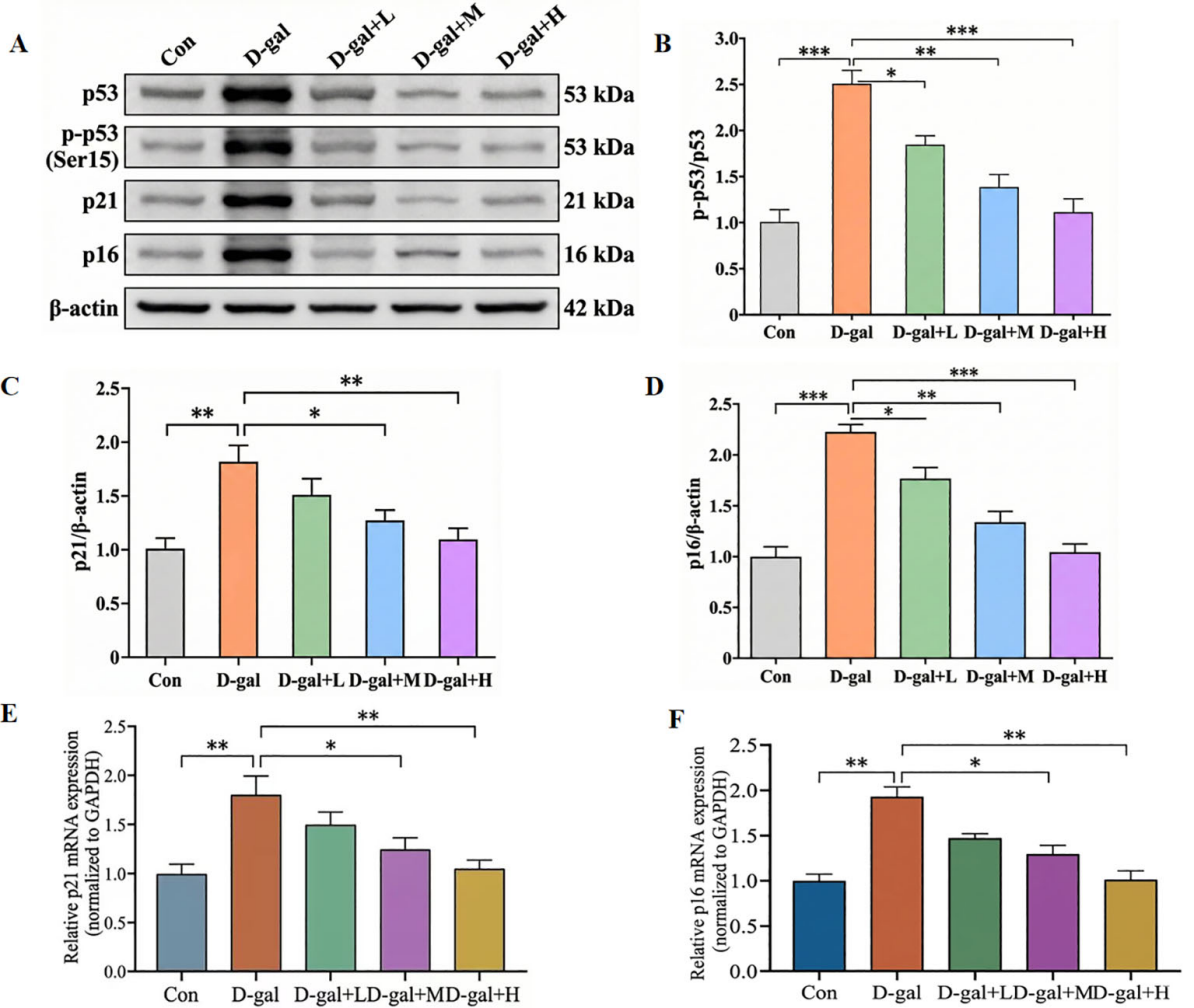
NaB inhibits the p53/p21/p16 signaling pathway in D-gal-induced senescent WI-38 cells. **(A)** Representative Western blot images of p53, p-p53 (Ser15), p21, and p16. β-actin served as loading control. **(B)** Quantification of p-p53/p53 ratio. **(C)** Quantification of p21/β-actin. **(D)** Quantification of p16/β-actin. **(E)** Relative p21 mRNA expression normalized to GAPDH, assessed by RT-qPCR. **(F)** Relative p16 mRNA expression normalized to GAPDH, assessed by RT-qPCR. Data are presented as mean ± SD (n = 3). *P < 0.05, **P < 0.01, ***P < 0.001.

The p53/p21/p16 signaling pathway constitutes a central molecular mechanism governing cellular senescence (Serrano et al., 1993; López-Otín et al., 2023; Huang et al., 2024; Yan et al., 2024). Given that oxidative stress can activate this pathway and NaB demonstrated significant ROS-scavenging capacity, we next examined whether NaB affects the p53 signaling cascade. Key proteins in the p53 pathway were analyzed by Western blot. D–gal treatment robustly increased total p53 and its phosphorylation at Ser15 (p–p53) (P < 0.001), upregulated the downstream target p21 (P < 0.01), and elevated the senescence marker p16 (P < 0.01; **Figures 4A–D**). NaB co–treatment dose–dependently suppressed p53 phosphorylation and reduced p21 and p16 expression. In the high–dose NaB group (D–gal+H), the p–p53/p53 ratio, p21, and p16 levels were restored to near–control values and differed significantly from the D–gal group (P < 0.001). These results indicate that NaB attenuates cell–cycle arrest and senescence progression by inhibiting the p53/p21/p16 signaling cascade. To further investigate whether NaB regulates p21 and p16 at the transcriptional level, RT-qPCR was performed to quantify their mRNA expression. As shown in **Figures 4E, F**, D-gal treatment significantly upregulated p21 mRNA (P < 0.01) and p16 mRNA (P < 0.01) compared with the control group. NaB co-treatment dose-dependently reduced the mRNA levels of both genes, with the high-dose NaB group (D-gal+H) showing significant reductions in p21 mRNA (P < 0.01 vs D-gal) and p16 mRNA (P < 0.01 vs D-gal), consistent with the observed decreases in protein expression.

### 3.5 NaB inhibits HDAC activity and restores histone acetylation levels in D-gal-induced senescent WI-38 cells

**Figure 5:**
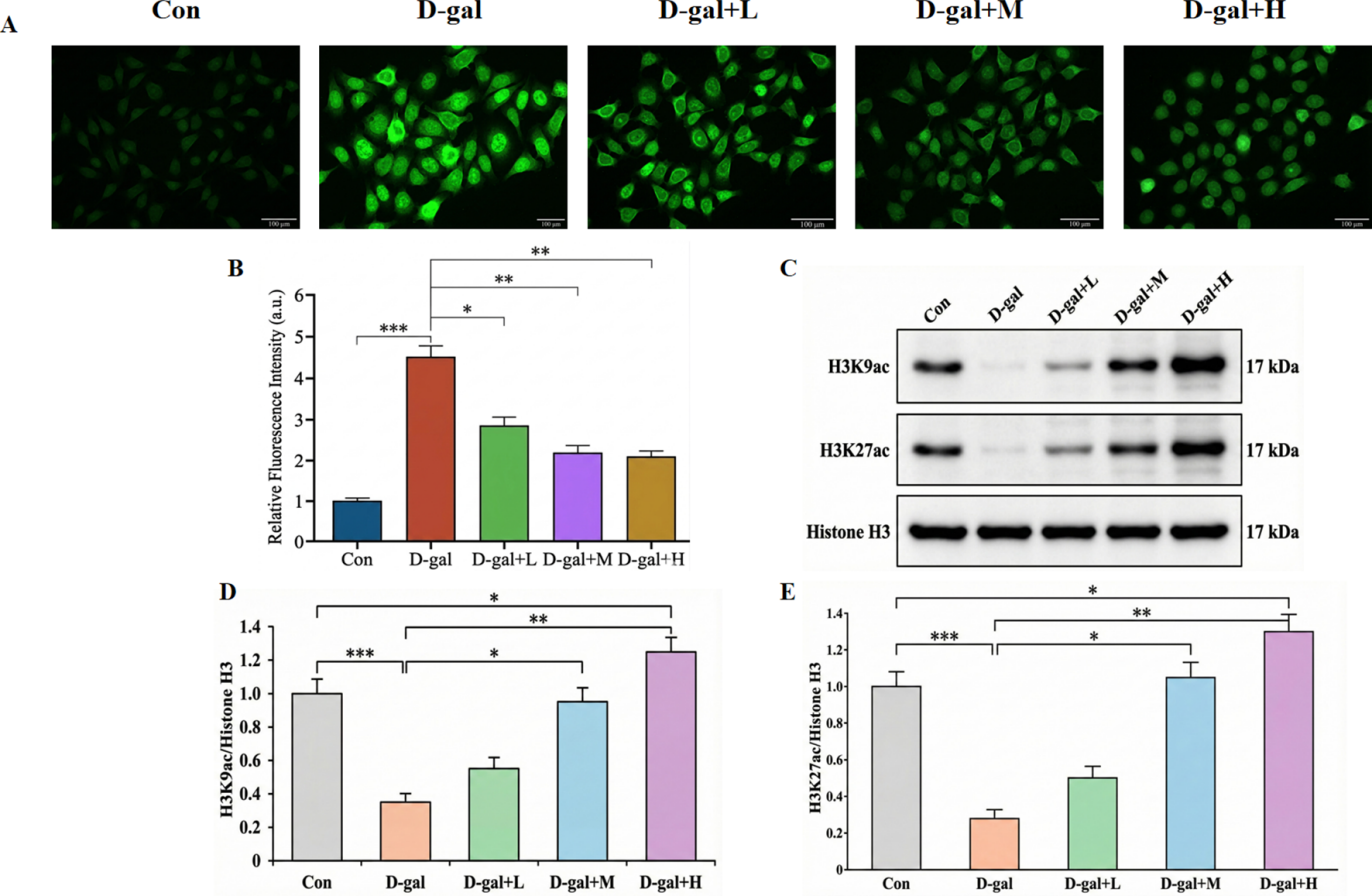
NaB inhibits HDAC activity and restores histone acetylation levels in D-gal-induced senescent WI-38 cells. **(A)** Representative fluorescence images of intracellular HDAC activity detected by HDAC activity fluorescent probe. Green fluorescence intensity positively correlates with HDAC activity. Scale bar = 100 μm. **(B)** Quantification of relative fluorescence intensity of HDAC activity. **(C)** Representative Western blot images of H3K9ac and H3K27ac protein expression. Histone H3 served as the loading control. **(D)** Quantification of H3K9ac/Histone H3. **(E)** Quantification of H3K27ac/Histone H3. Data are presented as mean ± SD (n = 3). *P < 0.05, **P < 0.01, ***P < 0.001.

NaB is a well-established class I/II HDAC inhibitor (Davie, 2003; Louis and Flint, 2017; Liu et al., 2018; McIntyre et al., 2019), and epigenetic dysregulation has been implicated in cellular senescence (Sen et al., 2016; McIntyre et al., 2019; Lee et al., 2022). To clarify NaB’s regulatory effects on epigenetic modifications in senescent cells, HDAC activity in each group was first detected using an HDAC activity fluorescent probe (**Figure 5A**). Results showed that Con group cells exhibited only weak green fluorescence, while the nuclear region of D-gal group cells displayed significantly enhanced green fluorescence intensity, suggesting markedly elevated HDAC activity in senescent cells. Following NaB intervention, fluorescence intensity decreased in a dose-dependent manner, with fluorescence intensity in the D-gal+H group approaching that of the Con group, indicating that NaB effectively inhibited D-gal-induced HDAC activity elevation.

Western blot results further confirmed (**Figures 5B–D**) that D-gal treatment led to significantly reduced H3K9ac and H3K27ac levels (P < 0.001 vs Con), indicating histone hypoacetylation status in senescent cells. As an HDAC inhibitor, NaB dose-dependently restored H3K9ac and H3K27ac levels. Compared with the D-gal group, H3K9ac and H3K27ac levels in the D-gal+M group were significantly elevated (P < 0.05), and both histone acetylation marks in the D-gal+H group recovered to control group levels (P < 0.01). These results suggest that NaB-mediated HDAC inhibition is associated with the regulation of senescence-related gene expression at the epigenetic level, as evidenced by the restoration of histone acetylation levels.

### 3.6 Nrf2 knockdown reverses NaB’s protective effects against D-gal-induced cellular senescence

**Figure 6:**
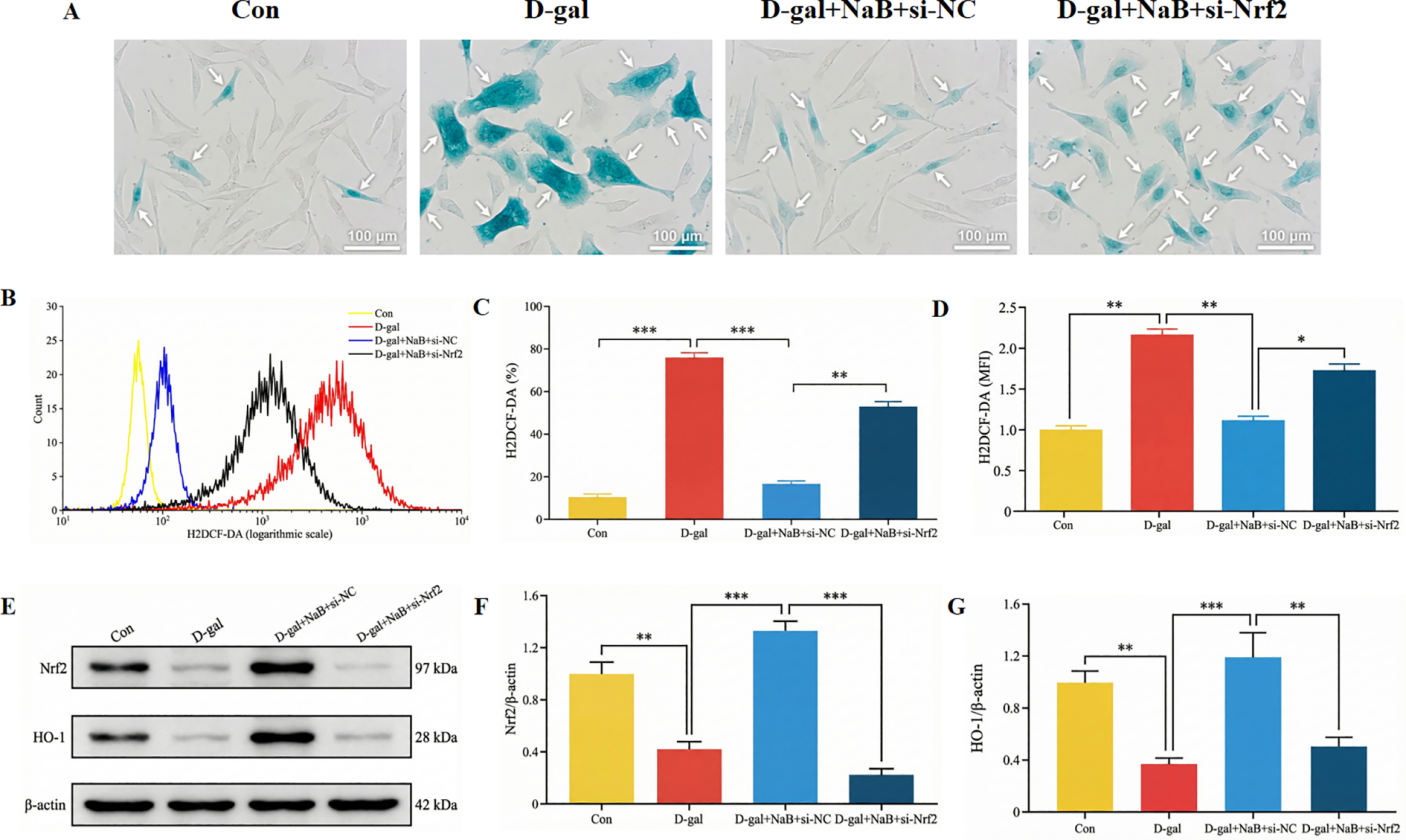
Nrf2 knockdown reverses the effects of NaB against D-gal-induced cellular senescence in WI-38 cells. WI-38 cells were transfected with si-NC or si-Nrf2, followed by co-treatment with D-gal (40 mM) and NaB (4 mM) for 72 (h) **(A)** Representative images of SA-β-Gal staining. White arrows indicate SA-β-Gal positive cells. Scale bar = 100 μm. **(B)** Flow cytometry analysis of intracellular ROS levels using H2DCF-DA probe. **(C)** Quantification of ROS-positive cell percentage. **(D)** Mean fluorescence intensity (MFI) of H2DCF-DA. **(E)** Representative Western blot images of Nrf2 and HO-1. β-actin served as loading control. **(F)** Quantification of Nrf2/β-actin. **(G)** Quantification of HO-1/β-actin. Data are presented as mean ± SD (n = 3). *P < 0.05, **P < 0.01, ***P < 0.001.

The above results demonstrated that NaB can activate the Nrf2 pathway. To further verify the causal relationship rather than mere correlation of the Nrf2 pathway in NaB’s anti-aging effects, siRNA technology was employed to knockdown Nrf2 expression. Western blot results showed (**Figures 6E, F**) that compared with the D-gal+NaB+si-NC group, Nrf2 protein expression was significantly reduced in the D-gal+NaB+si-Nrf2 group (P < 0.001), with knockdown efficiency of approximately 75%, confirming successful siRNA transfection.

SA-β-Gal staining results showed (**Figure 6A**) that cells in the D-gal+NaB+si-NC group displayed near-normal morphology with a low proportion of SA-β-Gal-positive cells; however, the proportion of positive cells significantly increased in the D-gal+NaB+si-Nrf2 group, with cells exhibiting obvious flattening and volume enlargement characteristic of the senescence phenotype. Flow cytometry results for ROS levels showed (**Figures 6B–D**) that the ROS-positive cell proportion (approximately 17%) and mean fluorescence intensity (MFI) in the D-gal+NaB+si-NC group were close to Con group levels; however, after Nrf2 knockdown, the ROS-positive rate in the D-gal+NaB+si-Nrf2 group significantly increased to approximately 52% (P < 0.01 vs si-NC group), and MFI was also markedly elevated (P < 0.05), indicating that NaB’s ROS-scavenging capacity was significantly impaired.

Further examination of Nrf2 downstream target gene HO-1 expression levels (**Figures 6E, G**) showed that HO-1 expression was significantly downregulated after D-gal treatment; NaB intervention could upregulate HO-1 expression (D-gal+NaB+si-NC group), while NaB’s upregulation of HO-1 was significantly blocked after Nrf2 knockdown (P < 0.01 vs si-NC group). These results demonstrate that Nrf2 knockdown partially reverses NaB’s protective effects against D-gal-induced cellular senescence, suggesting that Nrf2 activation contributes substantially, though not exclusively, to NaB’s anti-senescence activity.

### 3.7 NaB restores histone acetylation levels at p21 and p16 promoter regions in D-gal-induced senescence

The preceding results showed that NaB can inhibit HDAC activity and restore global histone acetylation levels while reducing p21 and p16 protein expression. To investigate whether NaB directly regulates histone acetylation status at p21 and p16 gene promoter regions through epigenetic mechanisms, ChIP-qPCR was performed to detect H3K9ac enrichment at the promoter regions of p21 (CDKN1A) and p16 (CDKN2A) genes.

**Figure 7:**
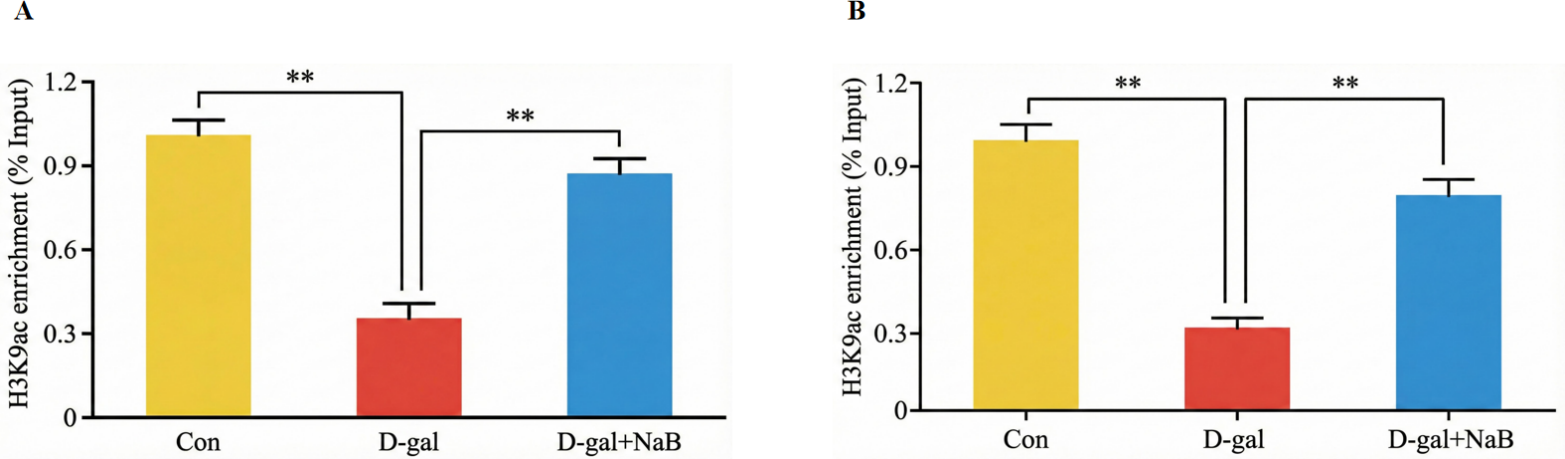
NaB restores H3K9ac enrichment at p21 and p16 promoter regions in D-gal-induced senescent WI-38 cells. ChIP-qPCR was performed to detect H3K9ac enrichment at the promoter regions of senescence-associated genes. **(A)** H3K9ac enrichment at the p21 (CDKN1A) promoter region. **(B)** H3K9ac enrichment at the p16 (CDKN2A) promoter region. Results are expressed as % Input. Data are presented as mean ± SD (n = 3). **P < 0.01.

Results showed (**Figure 7A**) that compared with the Con group, H3K9ac enrichment at the p21 promoter region was significantly reduced after D-gal treatment (P < 0.01), at approximately 32% of the Con group, suggesting histone hypoacetylation status at the p21 promoter region in senescent cells. Following NaB intervention, H3K9ac enrichment at the p21 promoter region was significantly restored (P < 0.01 vs D-gal group), reaching approximately 90% of the Con group. Similarly, H3K9ac enrichment at the p16 promoter region showed the same trend (**Figure 7B**). H3K9ac enrichment at the p16 promoter region in the D-gal group was significantly reduced compared to the Con group (P < 0.01), while NaB treatment significantly restored its acetylation level (P < 0.01 vs D-gal group).

These results indicate that D-gal-induced cellular senescence is accompanied by reduced histone acetylation levels at p21 and p16 promoter regions, while NaB, as an HDAC inhibitor, can participate in regulating senescence-related gene expression at the epigenetic level by inhibiting HDAC activity and restoring H3K9ac levels at p21 and p16 promoter regions. Combined with previous Western blot results (showing downregulation of p21 and p16 protein expression), this suggests that NaB’s regulation of senescence-related genes involves complex multi-level mechanisms, and the specific regulatory network warrants further investigation.

## 4 Discussion

This study systematically investigated the effects of sodium butyrate (NaB) on D–gal–induced senescence in WI–38 fibroblasts. The principal finding was that NaB exerted its modulatory action through three synergistic mechanisms: activation of the Nrf2/ARE antioxidant pathway, inhibition of the p53/p21/p16 senescence–signaling axis, and modulation of HDAC–mediated epigenetic regulation.

We first confirmed that NaB effectively attenuates D–gal–induced senescence phenotypes. SA–β–Gal, a canonical senescence marker whose elevated activity reflects lysosomal dysfunction and aging progression (Dimri et al., 1995), was dose–dependently reduced by NaB, accompanied by improved cellular morphology. Oxidative stress is a major driver of senescence, and excessive ROS can directly damage macromolecules and trigger the senescence program (Davalli et al., 2016). Consistent with this, D–gal treatment elevated ROS and MDA levels while suppressing SOD and GSH–Px activities, indicating redox imbalance. NaB treatment reversed these changes, scavenging ROS, lowering lipid peroxidation, and restoring antioxidant enzyme function—aligning with previous reports on its ability to modulate cellular redox status (Liu et al., 2018; Xu et al., 2018). Moreover, NaB suppressed D–gal–induced secretion of the pro–inflammatory cytokines IL–6 and IL–1β, thereby attenuating the SASP, which is crucial for limiting paracrine senescence and maintaining tissue homeostasis (Tchkonia et al., 2013).

Based on NaB’s known properties as an HDAC inhibitor and the established roles of oxidative stress and epigenetic regulation in senescence, we investigated three potential mechanistic pathways: the Nrf2/ARE antioxidant pathway, p53 signaling, and HDAC-mediated epigenetic regulation. Nrf2 is a master regulator of oxidative stress response. Under basal conditions, it is sequestered by Keap1 and targeted for degradation; upon stress, Nrf2 translocates to the nucleus and transactivates antioxidant genes such as HO–1 and NQO1 (Ngo and Duennwald, 2022). Age–related decline in Nrf2 activity has been linked to accelerated aging, whereas modulation of Nrf2 activity delays both cellular and organismal aging (Kubben et al., 2016; Medoro et al., 2024). Our data show that D–gal upregulated Keap1, reduced nuclear Nrf2, and downregulated HO–1 and NQO1. NaB reversed these effects, downregulating Keap1, promoting Nrf2 nuclear accumulation, and restoring HO–1/NQO1 expression. Critically, siRNA–mediated Nrf2 knockdown abolished NaB’s effects, as evidenced by increased SA–β–Gal positivity, elevated ROS, and failure to induce HO–1. These findings are consistent with a causal role for Nrf2 in NaB’s anti-senescence mechanism, echoing reports that highlight Nrf2 as a key regulator in premature aging (Kubben et al., 2016).

The p53/p21/p16 pathway is a central regulator of senescence (Huang et al., 2024). Oxidative stress or DNA damage activates p53, leading to p21 upregulation and G1/S arrest (Yan et al., 2024). Independently, p16 inhibits CDK4/6, maintaining Rb in a hypophosphorylated state and reinforcing cell–cycle exit (Serrano et al., 1993). We found that D–gal increased p53 phosphorylation and upregulated p21 and p16, whereas NaB dose–dependently suppressed p53 activation and reduced p21/p16 expression. Interestingly, crosstalk exists between the p53 and Nrf2 pathways: p53 can directly repress Nrf2 transcription, while Nrf2 activation may indirectly dampen p53 signaling by reducing ROS (Kopacz et al., 2020). NaB’s coordinated modulation of both pathways likely stems from its upstream attenuation of oxidative stress.

Epigenetic dysregulation is increasingly recognized in senescence. HDACs remove acetyl marks from histones, promoting chromatin compaction and gene repression (Lee et al., 2022). Senescent cells exhibit elevated HDAC activity and reduced acetylation at H3K9 and H3K27, contributing to the downregulation of cytoprotective genes (Sen et al., 2016). As a class I/II HDAC inhibitor, NaB elevates histone acetylation (Davie, 2003; McIntyre et al., 2019). We confirmed that D–gal increased HDAC activity and decreased H3K9ac/H3K27ac, while NaB inhibited HDAC function and restored acetylation levels. Importantly, ChIP–qPCR revealed that D–gal reduced H3K9ac enrichment at the promoters of p21 and p16, and NaB restored it. This indicates that NaB modulates senescence–related genes epigenetically by reinstating promoter acetylation.

Notably, NaB simultaneously lowered p21/p16 protein levels while increasing H3K9ac at their promoters. Although heightened acetylation is generally associated with transcriptional activation, this apparent paradox may be reconciled by several non-mutually exclusive mechanisms. First, in D-galactose-treated senescent cells, H3K9 hypoacetylation may reflect an aberrant, pathological chromatin state rather than a physiological silencing mechanism; restoration of H3K9ac by NaB may therefore represent normalization of chromatin dynamics rather than direct transcriptional activation per se. Second, NaB’s concurrent inhibition of p53 phosphorylation, the primary transcriptional activator of p21, may override any acetylation-mediated transcriptional enhancement, resulting in net downregulation of p21 mRNA and protein. Under this model, the dominant regulatory input determining p21/p16 expression is the p53 signaling axis rather than local histone acetylation status. Third, post-transcriptional mechanisms, including microRNA-mediated repression or enhanced protein turnover, cannot be excluded as contributing factors. HDAC inhibitors are well known to engage pleiotropic mechanisms (Xu et al., 2007), and the precise interplay between promoter acetylation and gene expression in this context warrants further investigation. It should be noted that the present data demonstrate an association between NaB-mediated HDAC inhibition and the observed epigenetic and phenotypic changes, rather than strict causality. Direct causal evidence would require rescue experiments such as HDAC overexpression to reverse NaB’s effects, or use of class-specific HDAC inhibitors to identify the relevant HDAC isoforms. These experiments represent important directions for future investigation.

Taken together, the three mechanisms identified in this study are not independent but form an integrated regulatory network. NaB, as a broad-spectrum HDAC inhibitor, directly restores histone acetylation at the chromatin level, creating a permissive epigenetic environment for the re-expression of cytoprotective genes. Simultaneously, the resulting increase in antioxidant gene transcription (via Nrf2/ARE activation) reduces the intracellular ROS burden, which in turn attenuates the oxidative stress-driven phosphorylation of p53 and downstream upregulation of p21 and p16. Thus, HDAC inhibition serves as an upstream epigenetic trigger that amplifies both the Nrf2 antioxidant response and the suppression of the senescence-signaling axis. This mechanistic convergence may explain the robust anti-senescence phenotype observed at higher NaB doses and suggests that the therapeutic efficacy of NaB in aging-related contexts is dependent on the coordinated action of these three pathways rather than any single mechanism in isolation.

The physiological significance of this work lies in several aspects. First, we systematically delineate how NaB modulates senescence via three integrated pathways, providing insights into the complex regulatory networks that govern cellular aging. Second, siRNA–based loss–of–function experiments established the necessity of Nrf2, moving beyond correlation to causation. Third, ChIP–qPCR uncovered an epigenetic layer of regulation, deepening our understanding of how HDAC inhibitors influence aging-related gene expression.

Several limitations should be noted. First, all experiments were conducted in WI-38 human fibroblasts *in vitro*, which limits the direct translational relevance of the findings. Since NaB is a gut microbiota-derived metabolite with systemic bioavailability, it’s *in vivo* pharmacokinetics, tissue distribution, and efficacy in organismal aging models remain to be established. Future studies should validate the current findings in established *in vivo* aging models such as D-galactose-treated rodents, which would allow assessment of NaB’s systemic effects on oxidative stress, epigenetic remodeling, and senescence-associated inflammation. Second, while Nrf2 was functionally validated, similar causal tests for the p53 and HDAC pathways (e.g., via p53 knockdown or HDAC overexpression) are warranted. Third, all experiments were performed in WI-38 human embryonic lung fibroblasts, a well-established model for replicative senescence studies. However, senescence mechanisms vary considerably across cell types, and the applicability of the current findings to primary fibroblasts, endothelial cells, neuronal cells, or other aging-relevant cell populations remains to be determined. WI-38 cells were selected in the present study due to their well-characterized response to D-galactose-induced oxidative stress and their wide use in senescence research; nevertheless, validation in additional cell models would substantially strengthen the generalizability of the conclusions. Fourth, some of the present findings are not without tension with existing literature. For instance, HDAC inhibitors have been reported to both promote and suppress senescence depending on cellular context and inhibitor specificity, and the net outcome may depend on the balance between pro-proliferative and pro-apoptotic gene expression changes. Additionally, the inclusion of a NaB-alone treatment group in future studies would provide useful within-study reference data to further distinguish the context-dependent effects of NaB from its intrinsic biological activity.

In conclusion, NaB protects against D–gal–induced cellular senescence by activating the Nrf2/ARE antioxidant pathway, inhibiting the p53/p21/p16 cascade, and modulating HDAC–dependent epigenetic marks. These findings contribute to our understanding of the molecular mechanisms underlying cellular senescence and highlight NaB as a useful tool for probing the physiological regulation of aging-related processes. Future work should evaluate NaB’s effects in animal models and explore its broader implications for understanding age-related physiological changes.

## 5 Conclusions

This study utilized a D–gal–induced WI–38 cellular senescence model to systematically explore the anti–aging effects of sodium butyrate (NaB) and its underlying mechanisms. The results demonstrate that NaB effectively mitigates D–gal–induced senescence phenotypes through coordinated modulation of multiple pathways. Specifically, NaB downregulates Keap1 expression, promotes Nrf2 nuclear translocation, and activates the transcription of downstream antioxidant genes HO–1 and NQO1, thereby enhancing cellular antioxidant defenses. Concurrently, NaB inhibits p53 phosphorylation and reduces the expression of p21 and p16, attenuating cell–cycle arrest. Furthermore, as an HDAC inhibitor, NaB restores global histone acetylation levels and modulates H3K9ac enrichment at the promoter regions of p21 and p16, implicating epigenetic regulation in its anti–senescence action. The causal involvement of these pathways was corroborated by siRNA–mediated Nrf2 knockdown and ChIP–qPCR experiments. The present study has several limitations, including reliance on a single *in vitro* cell model and the absence of *in vivo* validation. Future work should assess the anti–aging efficacy and safety of NaB in animal models, and examine its effects across different senescence contexts and cell types. In summary, these findings provide experimental support for NaB as a promising modulator of aging-related processes and offer novel mechanistic insights for the prevention and treatment of age–related diseases.

## Data Availability

The original contributions presented in the study are included in the article/supplementary material, further inquiries can be directed to the corresponding author/s.

## References

[B1] Baker D. J. Wijshake T. Tchkonia T. LeBrasseur N. K. Childs B. G. van de Sluis B. . (2011). Clearance of p16Ink4a-positive senescent cells delays ageing-associated disorders. Nature 479, 232–236. doi: 10.1038/nature10600. PMID: 22048312 PMC3468323

[B2] Childs B. G. Durik M. Baker D. J. van Deursen J. M. (2015). Cellular senescence in aging and age-related disease: from mechanisms to therapy. Nat. Med. 21, 1424–1435. doi: 10.1038/nm.4000. PMID: 26646499 PMC4748967

[B3] Coppé J. P. Desprez P. Y. Krtolica A. Campisi J. (2010). The senescence-associated secretory phenotype: the dark side of tumor suppression. Annu. Rev. Pathol. 5, 99–118. doi: 10.1146/annurev-pathol-121808-102144 20078217 PMC4166495

[B4] Davalli P. Mitic T. Caporali A. Lauriola A. D'Arca D. (2016). ROS, cell senescence, and novel molecular mechanisms in aging and age-related diseases. Oxid. Med. Cell. Longev. 2016, 3565127. doi: 10.1155/2016/3565127. PMID: 27247702 PMC4877482

[B5] Davie J. R. (2003). Inhibition of histone deacetylase activity by butyrate. J. Nutr. 133, 2485S–2493S. doi: 10.1093/jn/133.7.2485s. PMID: 12840228

[B6] Dimri G. P. Lee X. Basile G. Acosta M. Scott G. Roskelley C. . (1995). A biomarker that identifies senescent human cells in culture and in aging skin *in vivo*. Proc. Natl. Acad. Sci. U.S.A. 92, 9363–9367. doi: 10.1073/pnas.92.20.9363. PMID: 7568133 PMC40985

[B7] Geng Y. J. Wang Z. Zhou J. Y. Zhu M. G. Liu J. James T. D. (2023). Recent progress in the development of fluorescent probes for imaging pathological oxidative stress. Chem. Soc Rev. 52, 3873–3926. doi: 10.1039/d2cs00172a. PMID: 37190785

[B8] Hayflick L. Moorhead P. S. (1961). The serial cultivation of human diploid cell strains. Exp. Cell. Res. 25, 585–621. doi: 10.1016/0014-4827(61)90192-6. PMID: 13905658

[B9] He L. L. Yu W. T. Zhang W. X. Zhang L. (2021). An optimized two-step chromatin immunoprecipitation protocol to quantify the associations of two separate proteins and their common target DNA. STAR Protoc. 2, 100504. doi: 10.1016/j.xpro.2021.100504. PMID: 33997818 PMC8100613

[B10] Ho S. C. Liu J. H. Wu R. Y. (2003). Establishment of the mimetic aging effect in mice caused by D-galactose. Biogerontology 4, 15–18. doi: 10.1023/a:1022417102206. PMID: 12652185

[B11] Huang Y. Che X. Wang P. W. Qu X. (2024). p53/MDM2 signaling pathway in aging, senescence and tumorigenesis. Semin. Cancer Biol. 101, 44–57. doi: 10.1016/j.semcancer.2024.05.001. PMID: 38762096

[B12] Kopacz A. Kloska D. Forman H. J. Jozkowicz A. Grochot-Przeczek A. (2020). Beyond repression of Nrf2: An update on Keap1. Free Radic. Biol. Med. 157, 63–74. doi: 10.1016/j.freeradbiomed.2020.03.023. PMID: 32234331 PMC7732858

[B13] Kubben N. Zhang W. Q. Wang L. X. Voss T. C. Yang J. P. Qu J. . (2016). Repression of the antioxidant NRF2 pathway in premature aging. Cell 165, 1361–1374. doi: 10.1016/j.cell.2016.05.017. PMID: 27259148 PMC4893198

[B14] Kudlova N. De Sanctis J. B. Bhide M. (2022). Cellular senescence: Molecular targets, biomarkers, and senolytic drugs. Int. J. Mol. Sci. 23, 4168. doi: 10.3390/ijms23084168. PMID: 35456986 PMC9028163

[B15] Lee Y. Song M. J. Park J. H. Shin M. H. Kim M. K. Hwang D. . (2022). Histone deacetylase 4 reverses cellular senescence via DDIT4 in dermal fibroblasts. Aging (Albany NY) 14, 4653–4672. doi: 10.18632/aging.204118. PMID: 35680564 PMC9217707

[B16] Liu H. Wang J. He T. Becker S. Zhang G. Li D. . (2018). Butyrate: A double-edged sword for health? Adv. Nutr. 9, 21–29. doi: 10.1093/advances/nmx009. PMID: 29438462 PMC6333934

[B17] López-Otín C. Blasco M. A. Partridge L. Serrano M. Kroemer G. (2023). Hallmarks of aging: An expanding universe. Cell 186, 243–278. doi: 10.1016/j.cell.2022.11.001 36599349

[B18] Louis P. Flint H. J. (2017). Formation of propionate and butyrate by the human colonic microbiota. Environ. Microbiol. 19, 29–41. doi: 10.1111/1462-2920.13589. PMID: 27928878

[B19] McIntyre R. L. Daniels E. G. Molenaars M. Houtkooper R. H. Janssens G. E. (2019). From molecular promise to preclinical results: HDAC inhibitors in the race for healthy aging drugs. EMBO Mol. Med. 11, e9854. doi: 10.15252/emmm.201809854. PMID: 31368626 PMC6728603

[B20] Medoro A. Saso L. Scapagnini G. Davinelli S. (2024). NRF2 signaling pathway and telomere length in aging and age-related diseases. Mol. Cell. Biochem. 479, 2597–2613. doi: 10.1007/s11010-023-04878-x. PMID: 37917279 PMC11455797

[B21] Muthamil S. Kim H. Y. Jang H. J. Lyu J. H. Shin U. C. Go Y. . (2024). Biomarkers of cellular senescence and aging: Current state-of-the-art, challenges and future perspectives. Adv. Biol. (Weinh) 8, e2400079. doi: 10.1002/adbi.202400079. PMID: 38935557

[B22] Ngo V. Duennwald M. L. (2022). Nrf2 and oxidative stress: A general overview of mechanisms and implications in human disease. Antioxidants (Basel) 11, 2345. doi: 10.3390/antiox11122345. PMID: 36552553 PMC9774434

[B23] Sen P. Shah P. P. Nativio R. Berger S. L. (2016). Epigenetic mechanisms of longevity and aging. Cell 166, 822–839. doi: 10.1016/j.cell.2016.07.050. PMID: 27518561 PMC5821249

[B24] Serrano M. Hannon G. J. Beach D. (1993). A new regulatory motif in cell-cycle control causing specific inhibition of cyclin D/CDK4. Nature 366, 704–707. doi: 10.1038/366704a0. PMID: 8259215

[B25] Shwe T. Pratchayasakul W. Chattipakorn N. Chattipakorn S. C. (2018). Role of D-galactose-induced brain aging and its potential used for therapeutic interventions. Exp. Gerontol 101, 13–36. doi: 10.1016/j.exger.2017.10.029. PMID: 29129736

[B26] Tchkonia T. Zhu Y. van Deursen J. Campisi J. Kirkland J. L. (2013). Cellular senescence and the senescent secretory phenotype: therapeutic opportunities. J. Clin. Invest. 123, 966–972. doi: 10.1172/jci64098. PMID: 23454759 PMC3582125

[B27] Walsh M. E. Bhattacharya A. Sataranatarajan K. Qaisar R. Sloane L. Rahman M. M. . (2015). The histone deacetylase inhibitor butyrate improves metabolism and reduces muscle atrophy during aging. Aging Cell 14, 957–970. doi: 10.1111/acel.12387. PMID: 26290460 PMC4693467

[B28] Wang S. S. Zhang X. Ke Z. Z. Wen X. Y. Li W. D. Liu W. B. . (2022). D-galactose-induced cardiac ageing: A review of model establishment and potential interventions. J. Cell. Mol. Med. 26, 5335–5359. doi: 10.1111/jcmm.17580. PMID: 36251271 PMC9639053

[B29] Xu Y. H. Gao C. L. Guo H. L. Zhang W. Q. Huang W. Tang S. S. . (2018). Sodium butyrate supplementation ameliorates diabetic inflammation in db/db mice. J. Endocrinol. 238, 231–244. doi: 10.1530/joe-18-0137. PMID: 29941502

[B30] Xu W. S. Parmigiani R. B. Bhalla K. N. (2007). Histone deacetylase inhibitors: molecular mechanisms of action. Oncogene 26, 5541–5552. doi: 10.1038/sj.onc.1210620. PMID: 17694093

[B31] Yan J. Y. Chen S. Y. Yi Z. M. Zhao R. W. Zhu J. Y. Ding S. W. . (2024). The role of p21 in cellular senescence and aging-related diseases. Mol. Cells 47, 100113. doi: 10.1016/j.mocell.2024.100113. PMID: 39304134 PMC11564947

